# Impact of a Prehospital Chest Pain Alert App–Mediated Prehospital–in-Hospital Coordination Model on Treatment Delays and Clinical Outcomes in Patients With ST-Elevation Myocardial Infarction: Protocol for a 4-Year Retrospective Real-World Cohort Study

**DOI:** 10.2196/90144

**Published:** 2026-04-13

**Authors:** Shuyuan Chen, Rensong Wang, Jiangwei Ma, Weichun Xiao, Changxin Song

**Affiliations:** 1 Emergency Department, Shanghai Fengxian District Medical Emergency Center, No. 160 Deshun Road, Fengxian District, Shanghai, 201499, China, 86 16601809581; 2 Department of Cardiology, Fengxian District Central Hospital, Shanghai, China; 3 Department of Medical Education, Shanghai Fengxian District Medical Emergency Center, Shanghai, China; 4 School of Artificial Intelligence Application, Shanghai Urban Construction Vocational College, Shanghai, China

**Keywords:** ST-elevation myocardial infarction, STEMI, prehospital-hospital coordination, mobile health, mHealth, treatment delay, clinical outcome, real-world study, retrospective cohort study, alert app

## Abstract

**Background:**

The effectiveness of ST-elevation myocardial infarction (STEMI) treatment is highly time-dependent, and the information barrier between prehospital and in-hospital settings remains a key driver of treatment delays. Existing digital coordination tools either have a single function or lack long-term real-world evidence, making it difficult to meet clinical needs. This study adopts a prehospital chest pain alert app developed by the Fengxian District Medical Emergency Center. Mediated through a WeChat-based chest pain center group, the app enables prehospital information synchronization, real-time alerts, multidisciplinary coordination, and feedback on treatment outcomes to form a closed-loop model, overcoming the information barrier.

**Objective:**

This protocol aims to evaluate the impact of the app-mediated prehospital–in-hospital coordination model on treatment delays (eg, time from first electrocardiogram to catheterization laboratory preactivation and door-to-wire time) and clinical outcomes (eg, 30-day major adverse cardiovascular events, and 1-year and 4-year all-cause mortality) in patients with STEMI, and to assess its generalizability in high-risk subgroups.

**Methods:**

This is a single-center retrospective cohort study. Patients with STEMI admitted to Fengxian District Central Hospital from January 1, 2019, to December 31, 2024, will be enrolled and categorized into 3 groups: baseline group (January 1, 2019, to December 31, 2020, without app use), intervention group (January 1, 2021, to December 31, 2024, with app-mediated coordination), and concurrent control group (patients with STEMI who came to the hospital independently without calling an ambulance or were transported by ambulance but not reported via the app during the same period). The primary outcome is door-to-wire time. Secondary outcomes include other treatment delay indicators, clinical prognosis, and app operational efficiency. We will use propensity score matching to control for baseline confounding, segmented linear regression to analyze intervention trend effects, and subgroup analysis to assess generalizability in high-risk populations.

**Results:**

This study is based on 4 years of real-world data from the Department of Cardiology and the STEMI database of Fengxian District Central Hospital. As of April 2026, all 2019-2021 data have been collected; a sample size of 944 or more is expected. Data cleaning and statistical analysis are scheduled from May 2026 to June 2026.

**Conclusions:**

Based on 4 years of real-world data, combined with propensity score matching and interrupted time series analysis, this study aims to provide high-quality observational evidence for the app-mediated prehospital–in-hospital coordination model. The findings are anticipated to offer preliminary references for optimizing regional STEMI care systems and to inform the potential application of digital health technologies in acute coronary syndrome management.

## Introduction

ST-elevation myocardial infarction (STEMI) is a life-threatening acute cardiovascular event, and timely reperfusion therapy is crucial for improving prognosis [1]. Guidelines recommend a door-to-wire time (D2W) of 90 minutes or less for patients with STEMI in percutaneous coronary intervention (PCI)-capable centers and a transfer time of 120 minutes or less for those in non-PCI-capable centers transferred to PCI centers [2]. However, asynchronous prehospital and in-hospital information, delayed catheterization laboratory activation [3], and low efficiency of multidisciplinary coordination remain prevalent, leading to widespread treatment delays that significantly increase patient mortality and the risk of adverse outcomes [4]. Current guidelines and prior research highlight the urgency of optimizing prehospital–in-hospital coordination [5,6].

Existing digital intervention tools mostly focus on a single function (eg, electrocardiogram [ECG] transmission) and lack a full-chain design covering “information collection-real-time alert-coordinated disposal-feedback closed loop” [6]. Additionally, evidence regarding their generalizability in high-risk populations (eg, older adult patients and those with diabetes or hypertension) is insufficient. Previous small-scale pilots have shown that the prehospital chest pain alert app (hereafter referred to as the app) can shorten information transmission time, but its long-term real-world effectiveness remains unclear [7].

Leveraging 4 years of real-world data, this retrospective cohort study seeks to evaluate the impact of the app-mediated coordination model on treatment delays and clinical outcomes, as well as its generalizability in high-risk subgroups. The study aims to generate preliminary evidence to inform the optimization of regional STEMI care systems.

## Methods

### Study Design

This is a single-center retrospective cohort study. The study design, variable definitions, and statistical analysis plan are detailed below, and data collection and analysis will be strictly performed in accordance with this protocol without arbitrary changes to core designs. The study will be conducted at Fengxian District Central Hospital. Patients with STEMI diagnosed from January 1, 2019, to December 31, 2024, will be included and divided into 3 groups: baseline group (January 1, 2019-December 31, 2020, before the app was developed), intervention group (January 1, 2021-December 31, 2024, with app-mediated coordination), and concurrent control group (patients with STEMI who did not use the app during the same period, either self-admitted or transported by ambulance without app reporting). To mitigate potential confounding from temporal trends in STEMI care (eg, accumulated clinical experience of the care team and incremental workflow optimizations of the chest pain center) that might overestimate the app’s intervention effect, we included a concurrent control group consisting of patients with STEMI treated during the same period (2021‐2024) who did not use the app. This design allows us to compare the app-exposed group against contemporaneous, nonexposed patients, thereby helping to isolate the specific impact of the app intervention from broader system-wide improvements of the chest pain center.

### Prehospital Care Pathways

During the study period, all patients with STEMI were managed through 1 of 3 clinically distinct prehospital pathways, which also served as the basis for group classification in this study. The 3 pathways are as follows:

- Pathway A (self-presenting): patients arrive at the emergency department (ED) without using an ambulance. They must first undergo triage by the front-desk nurse; if chest pain is reported, they are immediately transferred to the chest pain center for in-hospital ECG and cardiac biomarker testing. Cardiology consultation is initiated only after abnormalities are confirmed.
- Pathway B (ambulance-transported, standard alert): patients are transported by ambulance but do not use the app. Prehospital personnel provide a verbal chest pain alert via traditional telephone, allowing the patient to bypass ED triage and proceed directly to the chest pain center for immediate in-hospital ECG upon arrival.
- Pathway C (ambulance-transported, app-mediated): patients are transported by ambulance, and the app is used. A prehospital 12-lead ECG is acquired at the scene and transmitted in real time to the hospital chest pain center via the app. If the on-duty cardiologist remotely confirms STEMI, the catheterization laboratory is preactivated before the patient leaves the scene. Upon hospital arrival, the patient bypasses the triage desk and is directly admitted to the chest pain center treatment area within the ED for rapid confirmation and precatheterization procedures (eg, informed consent). The ultimate goal of this pathway is to enable eligible patients to bypass the ED entirely and proceed directly to the catheterization laboratory (MultimediaAppendices 1  2).

By explicitly delineating these pathways, we identify the unique and irreplaceable core contributions of the app: real-time prehospital ECG acquisition and remote catheterization laboratory preactivation before patient arrival. These 2 mechanisms are the primary drivers of expected reductions in STEMI treatment delays and directly correspond to the novel door-to-preactivation time (D2P) indicator introduced in this study, which is designed to specifically capture the intervention effect of the app.

We acknowledge that the app-based intervention is a multicomponent integrated care model, encompassing prehospital 12-lead ECG acquisition, real-time data transmission, remote cardiologist diagnosis, and prehospital catheterization laboratory preactivation. This study is designed to evaluate the composite effect of this coordinated model on reducing STEMI treatment delays, while clarifying that the 2 unique and direct consequences of app use—early acquisition of a diagnostic ECG and subsequent remote catheterization laboratory preactivation—are the core drivers of delay reduction. Due to the inherent limitations of a retrospective cohort study design and the highly integrated nature of these components in real-world clinical practice, we are unable to disentangle the independent contribution of each individual component to the observed reduction in treatment delays.

We acknowledge that the baseline group (2019‐2020) and the concurrent control group (2021‐2024; non–app users) may inherently differ not only in app usage but also due to broader temporal trends, including the maturation of chest pain center protocols, accumulated clinical experience, and post-COVID adjustments in acute cardiac care. To mitigate this potential confounding, we used interrupted time series (ITS) analysis to quantify and separate the secular trend from the intervention effect. Additionally, the inclusion of a concurrent control group treated during the same period (2021‐2024) allows us to compare app-exposed patients with contemporaneous nonexposed patients, thereby partially isolating the app’s specific impact from system-wide improvements. Nevertheless, residual confounding cannot be fully excluded due to the retrospective design, and this limitation is acknowledged in the “Discussion” section.

### Study Population and Grouping

#### Inclusion Criteria

Patients were eligible for inclusion if they met all of the following criteria: (1) met STEMI diagnostic criteria (typical symptoms plus ST-segment elevation on ECG or elevated myocardial enzymes), (2) onset time of 12 hours or less, (3) aged 18 years or older, (4) had complete clinical data, and (5) underwent only forward conventional PCI as defined in the “Explanation for Limiting Surgical Methods: Rationale for Limitation” section [6,8].

#### Exclusion Criteria

Patients were excluded if they met any of the following criteria: (1) had end-stage diseases such as severe liver or kidney failure or malignant tumors; (2) refused PCI or abandoned treatment; (3) were interhospital-transferred patients (due to the unique characteristics of treatment delays in this population, requiring separate research [9]); (4) had severely missing clinical data; or (5) underwent nonforward conventional PCI, including but not limited to retrograde PCI, chronic total occlusion interventional therapy, coronary atherectomy, laser angioplasty, coronary artery bypass grafting combined with PCI, and simultaneous complex interventional therapy for multivessel disease (see “Explanation for Limiting Surgical Methods: Rationale for Limitation” section) [4,8].

#### Group Definitions

Patients with STEMI are categorized into 3 groups. First, the baseline group (January 2019 to December 2020) uses a conventional care model (only telephone notification and unable to transmit the first ECG). Second, the intervention group (January 2021 to December 2024) uses an app-mediated coordination model (prehospital emergency physicians upload patient information, the first ECG, and disposal measures via the app; the in-hospital team receives real-time alerts, preactivates the catheterization laboratory, and provides feedback on disposal suggestions). Finally, the concurrent control group (January 2021 to December 2024) consists of patients with STEMI not using the app (self-admitted or transported by ambulance without app reporting, ie, the first ECG was obtained within the hospital ED).

#### Sample Size Calculation

Referring to a systematic review by Gibson et al [6], the app intervention can shorten the mean D2W by 18.6 (SD 32.4) minutes. This study hypothesizes that the app can shorten D2W by 20 minutes, with an *α* level of .05 (2-sided) and a *β* of .10 (90% power). Using the sample size formula for a 3-arm design, each group requires 286 cases. Allowing for 10% attrition due to exclusion criteria, the total sample size needs to be 944 or more. Based on the center’s annual average of over 300 total chest pain or acute coronary syndrome admissions (with a mean of 260, SD 25 confirmed STEMI cases per year), the cumulative number of cases meets the requirement.

### Explanation for Limiting Surgical Methods: Rationale for Limitation

#### Clinical Dominance of Forward Conventional PCI

Forward conventional PCI is the most mainstream and standardized procedure for primary PCI in patients with STEMI. According to a systematic review by Gibson et al [6] and domestic STEMI diagnosis and treatment data, this procedure accounts for 85% to 90% of total STEMI-related PCI procedures. Its operational process is unified (forward approach puncture-angiography-balloon dilation-stent implantation) without additional complex technical interventions, making it an ideal research object for evaluating the impact of prehospital–in-hospital coordination models on treatment delays.

#### Avoiding Confounding Effects of Surgical Methods on Treatment Delays

Operational complexity and time consumption vary significantly among different PCI procedures: the average operation time for forward conventional PCI is 30 minutes to 1 hour [4], while that of complex procedures, such as chronic total occlusion intervention and retrograde PCI, can be 1.5 to 4 hours or longer [8]. The inclusion of multiple surgical methods will directly interfere with intergroup comparison of core outcome indicators, such as D2W and total ischemic time, leading to the true effect of the app-mediated coordination model being masked or exaggerated.

#### Focus on Core Research Objectives

The core goal of this study is to verify the improvement effect of the “app-mediated prehospital–in-hospital coordination model” on treatment delays and clinical outcomes, rather than comparing the advantages or effectiveness of different PCI procedures. By limiting the inclusion to patients undergoing forward conventional PCI, the time variation related to surgical operations can be homogenized, focusing the research on the process optimization effect of “information transmission–real-time alert–catheterization laboratory preactivation” and significantly improving the internal validity of the study results [6,10].

### Intervention Measures

#### Core Functions of the App

The app’s core functions include the following four aspects: (1) information collection—prehospital emergency personnel enter patient basic information, symptoms, vital signs, and 12-lead ECG images; (2) real-time transmission—AES-256 encrypted transmission supporting JPG or PNG format ECG upload, connected to the in-hospital chest pain center WeChat work group; (3) coordinated disposal—in-hospital cardiologists receive real-time alerts, remotely interpret ECGs, activate the catheterization laboratory, and provide feedback on disposal suggestions; and (4) closed-loop management—recording prehospital disposal (eg, dual antiplatelet drug use) and summarizing core in-hospital disposal to achieve a full-chain information closed loop [11].

#### Technical Parameters of the App

The technical parameters of the app are as follows: it was independently developed by the Fengxian District Medical Emergency Center; its operating platform supports iOS 12.0+ and Android 8.0+; and no commercial software copyright registration has been obtained for this software.

### Data Collection and Variable Definitions

#### Baseline Data

Including demographic characteristics (age, sex, and BMI), underlying diseases (hypertension, diabetes, and history of coronary heart disease), year of admission, and onset period (daytime or nighttime and weekday or holiday).

#### Treatment Delay Indicators

Treatment delay indicators include the time from the first ECG to catheterization laboratory activation; D2W, which, in reference to the 2025 American College of Cardiology/American Heart Association guidelines, is defined as the time from patient arrival at the emergency triage desk to guidewire passage through the infarct-related artery [1]; D2P, defined as the interval between patient arrival at the hospital door and the time of catheterization laboratory preactivation, where a negative value indicates that preactivation occurred before patient arrival—an outcome only achievable when a definitive STEMI diagnosis is made prehospital via real-time ECG transmission and early alert of the app. This metric directly captures the unique contribution of the app and is not confounded by secular trends (eg, staff experience and routine workflow changes), as such trends cannot transform a positive D2P value into a negative one; and total ischemic time, defined as the time from onset to guidewire passage.

To ensure consistency across the 2019‐2024 study period, all D2W data were extracted using the same standardized definitions: door time was defined as the patient’s arrival at the emergency triage desk (from electronic triage records), and wire time as the first guidewire passage (from PCI procedure logs). Extreme values were verified and handled according to prespecified rules. Thus, although the 2025 guidelines are cited for definitional clarity, the actual measurements are directly comparable throughout the study.

#### App Performance Metrics

To objectively reflect the real-world operational performance of the app (WeChat mini-program embedded), 3 core performance metrics were defined based on the clinical workflow of STEMI prehospital care, with all time and case data extracted from the app operation logs and chest pain center official records. The operational definitions and calculation methods were as follows. First, app STEMI alert response time was defined as the time interval (in minutes) from the first submission of prehospital ECG and STEMI alert information by prehospital medical staff via the app to the confirmation of receipt and issuance of catheterization laboratory preactivation instructions by the on-duty physician of the chest pain center via the app or coordination group. This was calculated for each individual case as the time difference between the app and clinical records, with the overall distribution summarized for reporting. Second, the timely and complete prehospital information submission rate within 5 minutes was defined as the proportion of STEMI cases in which prehospital medical staff completed the first timely and complete submission of core prehospital information (including the first ECG image, patient vital signs, onset time, and on-site preliminary diagnosis) via the app within 5 minutes of arriving at the patient’s side. This was calculated as (number of timely and complete submission cases/total number of app-based prehospital submission cases) × 100%. Finally, prehospital–in-hospital clinical information closed-loop feedback time was defined as the time interval (in hours) from the first prehospital information submission via the app to the post-PCI feedback of core in-hospital information (including angiographic results, surgical method, and postoperative blood flow status) by cardiologists via the app. This was calculated for each individual case as the time difference from the app logs, with the overall distribution summarized for reporting. All app performance metrics will be reported as descriptive statistics (mean, median, IQR, and percentage where applicable). These metrics are purely descriptive and will not be used to support any causal inferences regarding the app’s effect on STEMI treatment outcomes.

### Clinical Outcome Indicators

Clinical outcome indicators include short-term outcomes, which comprise 30-day major adverse cardiovascular events (including all-cause death, reinfarction, and heart failure) [12,13] and PCI success rate; long-term outcomes, which include 1-year and 4-year all-cause mortality [14,15] and left ventricular ejection fraction [16]; and app operational efficiency metrics, such as information transmission success rate [17,18].

### Data Sources

#### Overview

The data were mainly derived from the Data Reporting Platform of the Fengxian District Chest Pain Center and the background statistical data of the app. The former provides full-process timeline data including the sending time and response time of the first prehospital ECG, as well as key clinical information such as surgical approaches, subgroup classification, records of adverse events within 30 days, and 1-year and 4-year follow-up data. The latter compiles data on all patients treated through this collaborative system and calculates diagnostic accuracy. For this study, the data scope was strictly defined: we focused only on patients with STEMI managed within the system and excluded misdiagnosed patients and patients with non-STEMI, angina pectoris, and nonacute coronary syndrome.

#### Brief Description of Data Extraction and Coding Rules

A structured data extraction form (Multimedia Appendix 3) will be used to systematically collect all study variables. The form covers 5 modules: basic identification information, baseline clinical characteristics, treatment delay indicators, clinical outcomes, and app operational efficiency to ensure standardized and complete data collection. Data coding follows unified rules (Multimedia Appendix 3), clarifying the assignment of categorical variables, processing standards for continuous variables, and determination processes for missing and outlier values. All data will be extracted by 2 trained researchers using the double-blind method. After extraction, cross-validation will be performed and discrepancies will be adjudicated by a third cardiologist to ensure data accuracy.

### Statistical Analysis

#### Data Processing

Data will be extracted and verified by professional professors from local universities. EpiData 3.1 (EpiData Association) will be used to establish the database, and missing values will be handled by multiple imputation.

#### Statistical Methods

All statistical analyses were performed using Stata SE 17.0 (StataCorp LLC). Statistical analyses were planned as follows:

- Descriptive statistics: continuous variables will be expressed as mean (SD) or median (IQR), and categorical variables as n (%).
- Intergroup comparison: independent samples 2-tailed *t* test or rank sum test for continuous variables and chi-square test for categorical variables before propensity score matching (PSM), with matching variables including demographic characteristics (age, sex, and BMI), comorbidities (hypertension, diabetes, and history of coronary heart disease), and key care pathway characteristics (self-presenting vs ambulance-transported) to balance these factors between the baseline and concurrent control groups, and generalized linear model (normal distribution) or quantile regression (nonnormal distribution) after PSM.
- Time series analysis: segmented linear regression (ITS) will be used with the formula Yt = β0 + β1 × time + β2 × intervention + β3 × time × intervention + εt, with autocorrelation tested (Durbin-Watson test) and corrected using the autoregressive integrated moving average model (1,0,0) if needed [10].
- Survival analysis: Cox proportional hazards regression model will be used to calculate the adjusted hazard ratio and 95% CI.
- Subgroup analysis: stratification by age (≥65 y vs <65 y) [8], diabetes (yes or no) [19], and hypertension (yes or no) [20] to test interaction effects.

### Sensitivity Analysis

Given the inherent clinical heterogeneity of the concurrent control group—which comprises 2 distinct patient populations: (1) self-presenting patients with STEMI who must first undergo triage at the emergency triage desk before entering the chest pain center treatment area, and (2) ambulance-transported patients with STEMI not using the app who, via traditional telephone notification, bypass the triage desk and proceed directly to the chest pain center treatment area—these 2 subgroups have clear differences in baseline clinical characteristics and prehospital care pathways. Notably, the triage process itself directly adds time from hospital arrival to entry into the chest pain center treatment area for self-presenting patients. We will conduct a prespecified sensitivity analysis stratifying this group into these 2 subgroups to assess whether the app’s effect on core treatment delay indicators differs between these clinically distinct pathways and to evaluate the robustness of our primary findings against potential selection bias arising from these well-defined prehospital pathways and time-delay differences. It is important to note that the number of ambulance-transported patients not using the app in the later study period is extremely limited, as prehospital notification for STEMI cases was changed from “encouraged” to “mandatory” and incorporated into performance indicators after app implementation (with slightly more such cases only in the first year due to initial low compliance). Therefore, the statistical power of analyses focusing on this subgroup may be limited.

### Ethical Considerations

#### Ethical Approval

The study was approved by the medical ethics committee of Fengxian District Central Hospital (2025-KY-116‐02; January 14, 2026; Multimedia Appendix 4). Due to the use of retrospective deidentified data, informed consent was waived in accordance with the “Measures for the Ethical Review of Biomedical Research Involving Humans” issued by the National Health Commission of the People’s Republic of China [21]. No financial compensation was provided to participants in this retrospective study, as all data used were deidentified and retrospectively collected without direct participant interaction. All procedures comply with the Declaration of Helsinki and relevant national laws.

#### Data Security

For data security, strict protective measures are implemented in 3 key aspects involving deidentification standards, storage and access management, and data backup protocols. Only the last 4 digits of patients’ ID numbers are retained, their names are replaced with unique codes, and personal addresses are not recorded. Deidentified data are stored on encrypted servers with hierarchical authorized access, where the principal investigator can access the full dataset while statistical analysts only have access to coded data. In addition, a dual backup mechanism combined with regular off-site backups is adopted, and all data management practices are in full compliance with the Personal Information Protection Law of the People’s Republic of China and the Data Security Law of the People’s Republic of China.

### Quality Control

A comprehensive set of quality control measures is implemented for data processing, including outlier handling, consistency testing, and regular verification. For outlier handling, extreme values, such as a D2W exceeding 300 minutes, will be traced and verified, and invalid data caused by delayed recording will be excluded. For consistency testing, the double-blind method will be adopted for ECG interpretation, with any discrepancies in results adjudicated by a third independent expert. For regular verification, the completeness of collected data will be checked every 2 weeks to ensure the accuracy of data entry throughout the research process.

### Dissemination Plan

The findings of this study will be submitted to JMIR mHealth and uHealth for publication, focusing on the methodological framework of the novel D2P indicator and its clinical application in STEMI care. The practical experience of the prehospital–hospital coordinated treatment model will be shared at national academic conferences on emergency medicine and cardiology. Targeted practical training will also be provided for prehospital emergency personnel and chest pain center teams to promote the implementation of standardized treatment procedures. In addition, deidentified aggregated data will be shared in a public repository within 3 years after publication to facilitate methodological replication and secondary analysis by other researchers.

## Results

Study data will be obtained from eligible patients with STEMI admitted between January 1, 2019, and December 31, 2024. As of April 2026, all 2019-2021 data have been collected. Data cleaning and statistical analysis are scheduled from May 2026 to June 2026. This study will evaluate the impact of the app‑mediated prehospital–in-hospital coordination model on treatment delays and clinical outcomes in patients with STEMI, with baseline balance verification and subgroup analyses included.

## Discussion

### Overview

This study is a 4-year real-world retrospective cohort study designed to evaluate the effect of an app-mediated prehospital–in-hospital coordination model on STEMI treatment delays and clinical outcomes, as well as its generalizability in high-risk subgroups. We hypothesize that the primary findings of this study will be as follows: the app-mediated model will significantly reduce key treatment delay indicators, including D2W and the time from first ECG to catheterization laboratory activation, and the intervention group will exclusively present negative D2P values—a novel indicator that directly captures the app’s core effect of real-time prehospital ECG acquisition and remote catheterization laboratory preactivation before patient arrival. We also anticipate that the model will improve short-term clinical outcomes (eg, 30-day major adverse cardiovascular events) and reduce 1-year and 4-year all-cause mortality in patients with STEMI, with consistent beneficial effects in high-risk subgroups, including older adult patients (≥65 y) and those with hypertension or diabetes. Stratified sensitivity analysis of the concurrent control group is expected to verify the robustness of these primary findings, while the D2P indicator will effectively isolate the app’s intervention effect from confounding by secular trends in STEMI care (eg, accumulated staff experience and routine workflow optimizations). All interpretations of the study findings will be made with appropriate caution, and no causal claims will be overstated beyond the scope of the study’s methodology and real-world retrospective design.

### Principal Findings

The anticipated principal findings of this study center on the unique and irreplaceable core mechanisms of the app-mediated model in reducing STEMI treatment delays, which are clearly delineated by the 2 clinically distinct prehospital care pathways defined in this study: real-time prehospital 12-lead ECG acquisition and remote catheterization laboratory preactivation before patient hospital arrival. These 2 mechanisms are the direct and exclusive consequences of app use, and their intervention effect is specifically captured by the D2P indicator—negative D2P values in the intervention group will confirm that the app fundamentally alters the traditional STEMI care pathway by moving key diagnostic and catheterization laboratory activation steps from the in-hospital to the prehospital phase, a change that cannot be achieved by secular system-wide improvements of the chest pain center alone. The 3-arm cohort design (baseline, intervention, and concurrent control) is expected to further confirm that the observed reduction in treatment delays is attributable to the app intervention rather than temporal trends, as the concurrent control group (2021‐2024; nonapp users) allows for comparison with contemporaneous patients receiving standard care in the same mature chest pain center system (established since 2019 in our hospital). We also anticipate that the app-mediated model will show consistent effectiveness in high-risk STEMI subgroups, filling the evidence gap for digital health interventions in these clinically important populations. Furthermore, this study will confirm the composite effect of the app’s multicomponent design (information collection, real-time transmission, remote cardiologist diagnosis, and closed-loop management); due to the highly integrated nature of these components in real-world clinical practice and the inherent limitations of retrospective cohort design, the independent contribution of each individual component to treatment delay reduction cannot be disentangled in this study.

### Comparison With Prior Work

Existing research on digital health interventions for STEMI care has primarily focused on single-function tools (eg, standalone ECG transmission systems) and small-scale pilot studies with short follow-up periods, lacking long-term real-world evidence and full-chain design covering the entire prehospital–in-hospital coordination process [6,7]. A recent systematic review and meta-analysis by Gibson et al [6] found that smartphone apps can shorten the mean D2W by 18.6 minutes, but most included studies failed to account for temporal trend confounding or stratify heterogeneous control groups, leading to the potential risk of overattributing treatment delay reduction to digital interventions alone. In contrast, this study addresses these critical limitations by incorporating a concurrent control group and introducing the novel D2P indicator, which directly captures the app’s exclusive intervention effect and is not confounded by secular trends such as staff experience accumulation [1,3]. Additionally, previous studies have rarely evaluated the generalizability of digital interventions in high-risk STEMI subgroups [19,20], while this study specifically stratifies analysis by age, hypertension, and diabetes, providing targeted evidence for the clinical application of digital health tools in these populations. The app in this study also differs from existing digital intervention tools in its closed-loop management design, which integrates prehospital disposal records and in-hospital treatment feedback [11], aligning more closely with real clinical workflows and thus improving its practical applicability. Few studies have explicitly delineated prehospital care pathways to identify the core mechanisms of digital interventions for STEMI [22], and this study’s clear classification of 3 distinct pathways fills this gap by distinguishing the app’s unique contributions (pathway C) from standard care pathways (pathways A and B).

### Study Strengths

This study has 3 core strengths. First, the 3-arm cohort design combined with ITS analysis not only controls baseline confounding but also captures the long-term trend effect of the intervention with rigorous methodology. Second, the study focuses on high-risk subgroups, such as older adults and those with underlying diseases, thereby filling the evidence gap regarding digital interventions in this population. Third, the app is aligned with clinical workflows, includes clear technical details, ensures high reproducibility, and facilitates implementation.

A key strength of our study design is the introduction of the D2P as a novel process indicator. Unlike D2W, which can be influenced by numerous downstream factors (eg, PCI procedure complexity and in-hospital team efficiency), this indicator directly reflects the app’s core mechanism—enabling prehospital STEMI diagnosis and remote catheterization laboratory preactivation. This effect is exclusive to the app and cannot be confounded by secular trends such as staff experience accumulation or protocol evolution. The magnitude of the negative D2P value is directly dependent on the time elapsed from ambulance arrival at the ED door to patient presentation for care. We further enhanced the clarity of the app’s intervention mechanism by explicitly delineating 3 distinct prehospital care pathways and locking in the app’s unique core contributions, which are directly captured by the novel D2P indicator we introduced. The anticipated finding that a negative D2P value exists exclusively in the intervention group provides strong, direct evidence that the app fundamentally alters the STEMI care pathway in a way that cannot be explained by secular trends or system-wide improvements of the chest pain center. This metric thus serves as an internal validation of the app’s unique intervention effect.

### Study Limitations

Single-center data may limit external generalizability, and multicenter verification is needed in the future. Interhospital-transferred patients are not included. This population faces more prominent treatment delay issues, and the use of the app in this scenario needs further exploration [9]. The retrospective design may have residual confounding (eg, patient health care–seeking behavior), which has been minimized through PSM and stratified analysis. Additionally, the concurrent control group has inherent clinical heterogeneity, as it comprises 2 subgroups with distinct prehospital care pathways: self-presenting patients who require nurse-led front-desk triage before entering the chest pain center, and ambulance-transported patients without app use who directly enter the chest pain center via traditional telephone prenotification. These subgroups have distinct baseline clinical characteristics and prehospital care processes, and the nurse-led front-desk triage process directly increases the time from the patient’s hospital arrival to entry into the chest pain center treatment area, which introduces potential selection bias into the study. Although we have planned a prespecified sensitivity analysis to stratify the concurrent control group by prehospital pathways and verify the robustness of our primary findings, the statistical power of this stratified analysis is inherently limited: the sample size of ambulance-transported patients without app use is extremely small in the later study period, owing to the mandatory reporting requirement with assessment indicators for STEMI cases after app launch (a slightly larger sample only existed in the first year of app launch due to initial low usage compliance). This study evaluates the composite effect of the app-based multicomponent intervention model, and the inherent limitations of the retrospective cohort study design prevent us from disentangling the independent contribution of each individual component to treatment delay reduction. Residual confounding cannot be fully excluded due to the retrospective study design. Future prospective studies with more homogeneous control groups stratified by prehospital care pathways and larger sample sizes are therefore warranted to validate the conclusions of this study. Notably, the single-center design of this study is a result of the specific medical layout of Shanghai Fengxian District, where our hospital is the only institution with national chest pain center certification and emergency PCI qualification for STEMI treatment—this objective medical context limits the feasibility of a multicenter design in this study. It is critical to clearly distinguish between the generalizable core concept of the app-mediated model (prehospital real-time ECG transmission and remote catheterization laboratory preactivation, an evidence-based strategy recommended by international STEMI guidelines [1,2]) and the locally dependent implementation details (eg, the WeChat-based chest pain center group and the self-developed app); the former is universally applicable to STEMI care systems worldwide, while the latter can be adapted to local medical resources and communication tools. Furthermore, while the concurrent control group and D2P indicator minimize the risk of overattributing effects to the app, the intervention period overlaps with ongoing refinements of chest pain center protocols, accumulated clinical experience of the care team, and post-COVID operational adjustments in acute cardiac care, and part of the observed improvement in treatment delays may reflect broader system maturation rather than the app alone. Furthermore, the baseline group (2019‐2020) and the concurrent control group (2021‐2024; nonapp users) may inherently differ due to broader temporal trends (eg, COVID-19 impact and system-wide protocol evolution), which cannot be fully eliminated despite the use of ITS analysis and a concurrent control group design. This residual confounding is an inherent limitation of retrospective observational studies. All study conclusions will be restricted to the scope of the real-world retrospective design, and no overgeneralization to other medical settings or populations will be made.

### Future Directions

Future research could pursue 3 interconnected directions centered on the D2P indicator and app-mediated model. Starting with inward refinement, the determinants of D2P magnitude—including prehospital, in-hospital, and patient-specific factors—warrant further investigation to optimize ED bypass workflows, with standardized subnode decomposition of D2P’s calculation facilitating cross-institutional replication. Moving to outward generalization, multicenter studies are needed to validate context-specific D2P reference ranges and the model’s core mechanisms (prehospital ECG transmission and remote catheterization laboratory preactivation) across diverse emergency medical service systems, while adapting implementation details to local resources and replicating the study’s statistical framework for methodological consistency. Complementing these efforts, technological advancement could streamline D2P-related workflows by integrating artificial intelligence–assisted ECG diagnosis [23] into the app—accelerating atypical STEMI identification and reducing false activations—while health economic evaluations should anchor on D2P as a key process indicator, linking its reduction to tangible outcomes (eg, shorter length of stay and lower major adverse cardiovascular events–related costs) to maintain alignment with the study’s core design.

## Supplementary material

10.2196/90144 Multimedia Appendix 1 Prehospital data collection interface of the chest pain alert app.

10.2196/90144 Multimedia Appendix 2 In-hospital collaborative communication interface (WeChat work group).

10.2196/90144 Multimedia Appendix 3 Data extraction form and coding rules.

10.2196/90144 Multimedia Appendix 4 Ethical approval letter of this study (2025-KY-116-02).

## Abbreviations

D2P: door-to-preactivation time
D2W: door-to-wire time
ECG: electrocardiogram
ED: emergency department
ITS: interrupted time series
PCI: percutaneous coronary intervention
PSM: propensity score matching
STEMI: ST-elevation myocardial infarction
