# Supplementary figures and images for: Impact of a Prehospital Chest Pain Alert App–Mediated Prehospital–in-Hospital Coordination Model on Treatment Delays and Clinical Outcomes in Patients With ST-Elevation Myocardial Infarction: Protocol for a 4-Year Retrospective Real-World Cohort Study
Source: JMIR Res Protoc. 2026 Apr 13;15:e90144. doi: 10.2196/90144 (PMC13075538; doi:10.2196/90144)

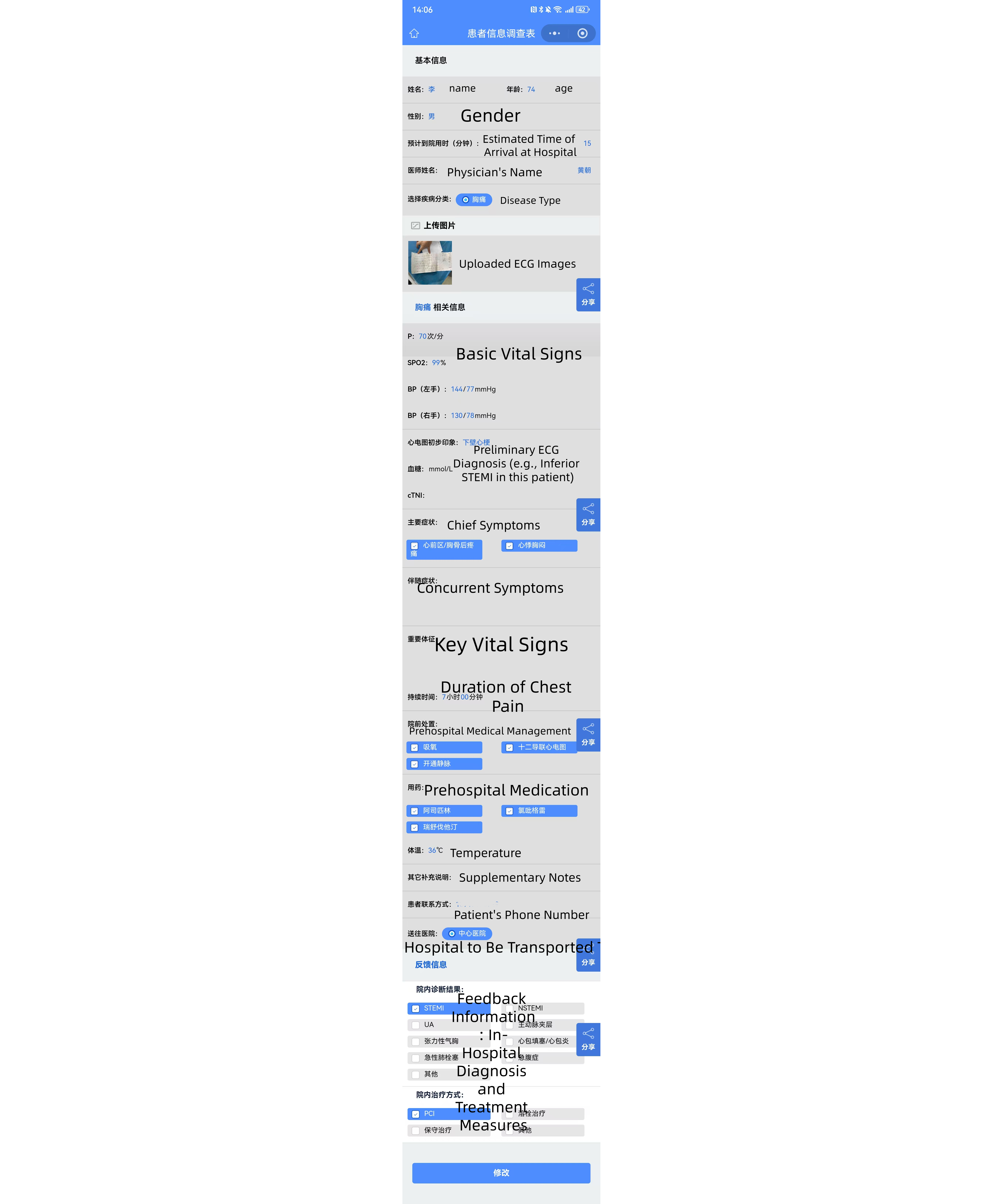

Supplement: Multimedia Appendix 1 [file resprot-v15-e90144-s001.png]

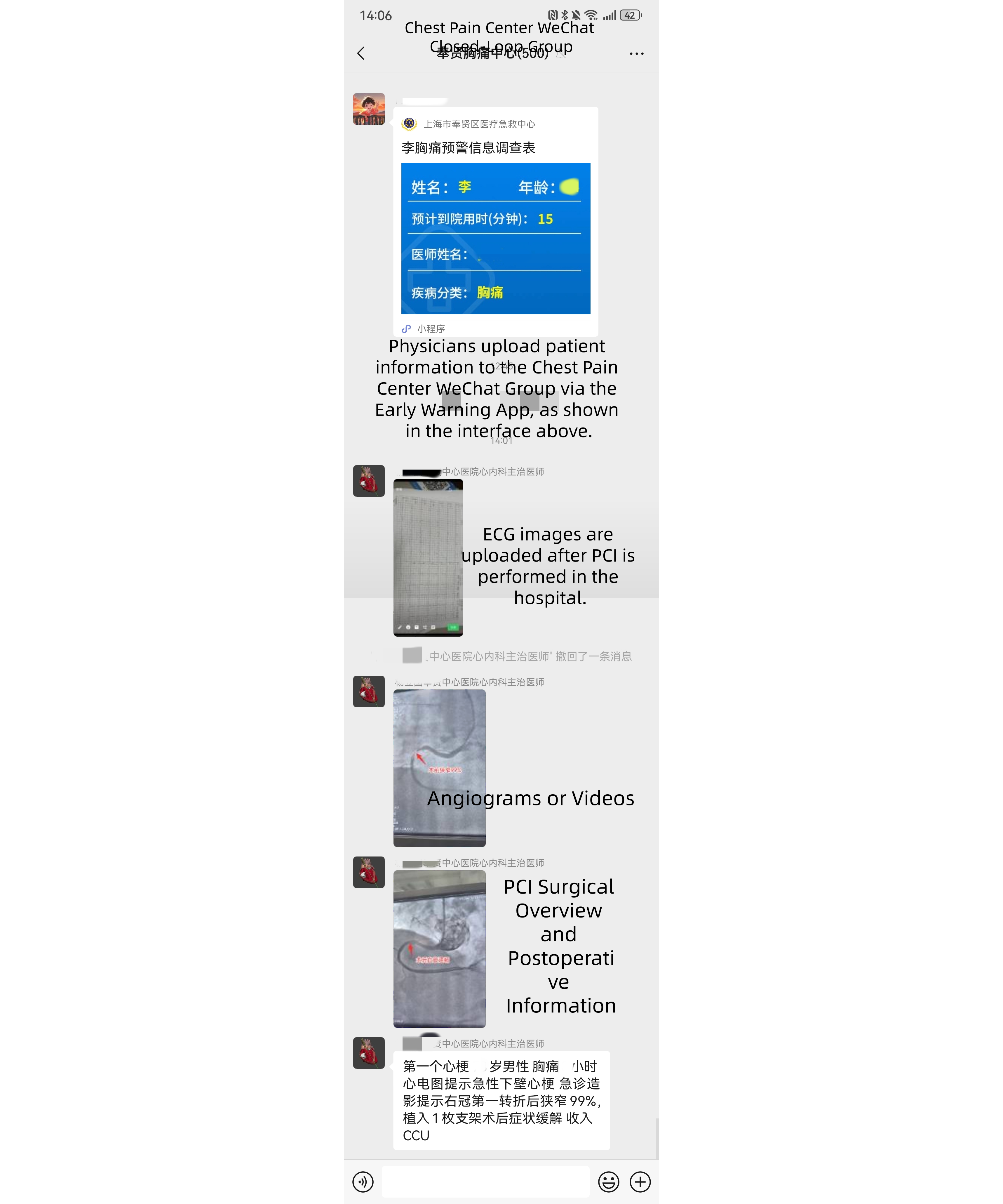

Supplement: Multimedia Appendix 2 [file resprot-v15-e90144-s002.png]
