# Supplementary material for: Impact of a Prehospital Chest Pain Alert App–Mediated Prehospital–in-Hospital Coordination Model on Treatment Delays and Clinical Outcomes in Patients With ST-Elevation Myocardial Infarction: Protocol for a 4-Year Retrospective Real-World Cohort Study
Source: JMIR Res Protoc. 2026 Apr 13;15:e90144. doi: 10.2196/90144 (PMC13075538; doi:10.2196/90144)
Supplement: Multimedia Appendix 3 [file resprot-v15-e90144-s003.docx]

**Appendix 1: Data Extraction Form and Coding Rules**

（Optimized & English Translated Version, Adapted for JMIR RP Submission）

**I. Data Extraction Form (Structured Table)**

**Module 1: Basic Identification Information (for Data Traceability and Grouping)**

| Variable Name | Variable Definition | Data Type | Data Source | Filling Instructions |
| --- | --- | --- | --- | --- |
| Unique Study ID | Formatted as "Year-Month-Sequence Number" (e.g., 201901001) to link all data of a single patient | Character | Researcher-defined (mapped to raw data) | Unique code; no duplicates allowed |
| HospitalizationNumber  /Visit ID | Unique patient identifier in the hospital’s electronic medical record (EMR) system | Character | Electronic Medical Record System, Fengxian District Central Hospital → "Patient Basic Information" → "Hospitalization Number" field | Accurate entry for medical record traceability |
| Group Type | Distinguishes baseline group, intervention group, and concurrent control group | Categorical | Study design + treatment records | Single selection; no multiple choices |
| Visit Date | Date of patient’s arrival at the emergency triage desk (accurate to day) | Date | Emergency triage record / EMR → "Visit Registration" → "Triage Date" field | Format: YYYY-MM-DD |
| Data Extractor | Name of the researcher responsible for extracting the patient’s data | Character | Researcher documentation | Facilitates tracing responsible personnel during quality audits |
| Data Verifier | Name of the researcher who reviewed the patient’s extracted data | Character | Researcher documentation | Double verification to ensure accuracy |

**Module 2: Baseline Clinical Characteristics (Aligned with Study Protocol’s "Baseline Data")**

| Variable Name | Variable Definition | Data Type | Data Source | Filling Instructions |
| --- | --- | --- | --- | --- |
| Gender | Patient’s biological gender | Categorical | EMR → "Demographic Information" → "Gender" field | Single selection: Male/Female |
| Age | Actual age of the patient at the time of visit (accurate to year) | Continuous | EMR → "Demographic Information" → "Date of Birth" field | Calculation: Visit year - birth year; extracted directly from medical records |
| Hypertension History | Pre-existing hypertension (including medication history) or hypertension diagnosed at admission | Categorical | EMR → "Past Medical History" + admission blood pressure record → "Vital Signs" field | Single selection: Yes/No |
| Diabetes Mellitus History | Pre-existing diabetes (including medication/insulin history) or diabetes diagnosed at admission | Categorical | EMR → "Past Medical History" + admission blood glucose record → "Laboratory Examinations" field | Single selection: Yes/No |
| Coronary Artery Disease (CAD) History | Pre-existing CAD (including prior PCI/CABG history) | Categorical | EMR → "Past Medical History" → "Cardiovascular Disease" field | Single selection: Yes/No |
| Onset Period (Day/Night) | Time period of symptom onset reported by the patient | Categorical | Emergency medical record / EMR → "Present Illness" → "Onset Time" field | Single selection: Day (06:00–18:00)/Night (18:00–06:00 next day) |
| STEMI ICD-10 Code | Primary discharge diagnosis code (compliant with single-disease quality monitoring requirements) | Character | EMR → "Discharge Diagnosis" → "Primary Diagnosis Code" field | Only select I21.0/I21.1/I21.2/I21.3/I21.9 |
| Body Mass Index (BMI) | BMI at the time of visit (weight/height²) | Continuous | EMR → "Physical Examination" → "Weight" and "Height" fields | Retain 1 decimal place; unit: kg/m²; code as "NA" if missing |
| Onset Date Type | Whether the onset date is a statutory working day | Categorical | Onset date + national statutory holiday list | Single selection: Working day (Monday–Friday, excluding adjusted rest days)/Holiday (including weekends and statutory holidays) |

**Module 3: Treatment Delay Indicators (Primary Outcomes, Aligned with Study Protocol)**

| Variable Name | Variable Definition | Data Type | Data Source | Filling Instructions |
| --- | --- | --- | --- | --- |
| Time of First ECG Completion | Time when the first 12-lead ECG was completed (prehospital or in-hospital emergency; accurate to minute) | Time | Emergency medical record / ECG report → "Report Time" field | Format: YYYY-MM-DD HH:MM |
| Catheterization Laboratory (Cath Lab) Preactivation Time | Time when the in-hospital team initiated the cath lab after receiving the alert (accurate to minute) | Time | Chest Pain Center Registry / App backend logs → "Alert Response Time" field | For the intervention group, link to App alert time |
| Time from First ECG to Cath Lab Activation | Interval between first ECG completion and cath lab preactivation | Continuous | Calculated (unit: minutes) | Mark as "Abnormal" for verification if negative |
| Emergency Department (ED) Arrival Time | Time when the patient arrived at the ED triage desk (accurate to minute) | Time | Emergency triage record → "Triage Time" field | Format: YYYY-MM-DD HH:MM |
| PCI Guidewire Crossing Time | Time when the guidewire first crossed the infarct-related artery during PCI (accurate to minute) | Time | PCI operation record / Chest Pain Center Registry → "STEMI-3.2" indicator | Aligned with single-disease quality metrics |
| Door-to-Wire Time (D2W) | Interval between ED arrival time and guidewire crossing time | Continuous | Calculated (unit: minutes) | Primary outcome; mark abnormal values (>300 minutes) for verification |
| Patient-Reported Onset Time | Time when symptoms first appeared (reported by the patient or family; accurate to minute) | Time | Emergency medical record / EMR → "Present Illness" → "Onset Time" field | Format: YYYY-MM-DD HH:MM; code as "Unknown" if unrecallable |
| Total Ischemic Time | Interval between patient-reported onset time and guidewire crossing time | Continuous | Calculated (unit: minutes) | Mark as "NA" if onset time is unknown |
| Cath Lab Response Time (Intervention Group Only) | Interval between App alert sending and in-hospital team’s confirmation of cath lab preactivation | Continuous | App backend logs + Chest Pain Center Registry | Calculation: Cath lab preactivation time - App alert time; unit: minutes |

**Module 4: Clinical Outcome Indicators (Short-Term/Long-Term + App Efficacy)**

| Variable Name | Variable Definition | Data Type | Data Source | Filling Instructions |
| --- | --- | --- | --- | --- |
| 30-Day MACE Occurrence | Occurrence of all-cause death, reinfarction, or heart failure within 30 days after PCI (per study protocol definition) | Categorical | Outpatient follow-up records + telephone follow-up + medical insurance database | Single selection: Not occurred/Occurred - All-cause death/Occurred - Reinfarction/Occurred - Heart failure |
| PCI Success Rate | Whether the infarct-related artery achieved TIMI 3 flow after PCI (Thrombolysis in Myocardial Infarction flow grade) | Categorical | PCI operation record → "Post-procedural Flow Grade" field | Single selection: Successful (TIMI 3)/Failed (TIMI 0–2); record failure reason (e.g., vessel occlusion, dissection) if failed |
| 1-Year All-Cause Mortality | Occurrence of all-cause death within 1 year after PCI | Categorical | Medical insurance database + telephone follow-up | Single selection: Yes/No; record death date (YYYY-MM-DD) if deceased |
| 4-Year All-Cause Mortality | Occurrence of all-cause death within 4 years after PCI | Categorical | Medical insurance database + outpatient follow-up | Single selection: Yes/No/Under follow-up; record death date if deceased |
| Left Ventricular Ejection Fraction (LVEF) | LVEF value within 1 week after PCI or before discharge | Continuous | Echocardiogram report → "Left Ventricular Function" → "LVEF" field | Retain 1 decimal place (e.g., 52.5%) |
| Information Transmission Success Rate (Intervention Group Only) | Whether App-uploaded information (including ECG) was complete and successfully received in-hospital (no loss/damage) | Categorical | App backend logs + manual verification | Single selection: Successful/Failed |
| Prehospital-In-Hospital ECG Diagnosis Consistency (Intervention Group Only) | Consistency between prehospital ECG interpretation (by emergency physicians via App) and in-hospital ECG interpretation (by cardiologists) | Categorical + Calculated | Emergency medical record + ECG report + Kappa value calculation | Single selection: Consistent/Inconsistent; Kappa ≥0.75=excellenconsistency, 0.4–0.75=moderate consistency, <0.4=poor consistency; resolve discrepancies via third expert adjudication |

**II. Coding Rules (Unified Data Entry Standards)**

**1. Coding Table for Categorical Variables (Core Variables)**

| Variable Category | Specific Options | Code Value | Notes (Supplementary Explanations) |
| --- | --- | --- | --- |
| Group Type | Baseline group (2019–2020) | 1 | Conventional treatment mode; no App use |
|  | Intervention group (2021–2024) | 2 | App-mediated prehospital-in-hospital coordination mode |
|  | Concurrent control group (2021–2024) | 3 | No App use (self-admitted or ambulance-transported without App reporting) |
| Gender | Male | 1 | — |
|  | Female | 0 | — |
| Hypertension History | Yes (pre-existing or diagnosed at admission) | 1 | Meets either: ① Prior diagnosis record; or ② Admission SBP ≥140 mmHg and/or DBP ≥90 mmHg (non-emergent state) |
|  | No | 0 | No prior history + normal admission blood pressure |
| Diabetes Mellitus History | Yes (pre-existing or diagnosed at admission) | 1 | Meets either: ① Prior diagnosis record; or ② Admission fasting blood glucose ≥7.0 mmol/L; or ③ Random blood glucose ≥11.1 mmol/L |
|  | No | 0 | No prior history + normal admission blood glucose |
| 30-Day MACE Occurrence | Not occurred | 0 | No all-cause death, reinfarction, or heart failure |
|  | Occurred - All-cause death | 1 | Record death date (accurate to day) |
|  | Occurred - Reinfarction | 2 | Confirm with ECG/myocardial enzyme recheck evidence |
|  | Occurred - Heart failure | 3 | Confirm with elevated BNP + echocardiogram/chest X-ray evidence |
| PCI Success Rate | Successful (TIMI 3) | 1 | Infarct-related artery achieves TIMI 3 flow |
|  | Failed (TIMI 0–2) | 0 | Record failure reason (e.g., vessel occlusion, dissection) |
| Information Transmission Success Rate | Successful | 1 | Complete patient information + readable ECG + successful in-hospital reception |
|  | Failed | 0 | Includes information loss, blurred ECG, or transmission timeout (>10 minutes) |
| Onset Date Type | Working day | 1 | Monday–Friday (excluding adjusted rest days) |
|  | Holiday | 0 | Weekends + national statutory holidays |

**2. Coding Rules for Continuous Variables**

- **Time-related variables**: Unified unit = minutes; calculated as "end time - start time". For negative results (e.g., recording errors), code as "888" and mark "Abnormal time record; trace and verify" in the "Remarks" column. Correct after verification; treat as missing if unverifiable.
- **Numeric variables** (e.g., age, BMI, LVEF): Retain 1 decimal place. For values outside the reasonable range (age >120 years, BMI >60 or <10, LVEF >80% or <20%), code as "888" and mark "Abnormal value; trace and verify" in the "Remarks" column. Correct after reviewing medical records.
- **Missing value handling**: Code missing categorical variables as "999" and missing continuous variables as "NA". Do not leave blank to facilitate multiple imputation using Epi Data 3.1.

**3. Coding Notes for Special Indicators**

- **STEMI ICD-10 Code**: Strictly extract the primary discharge diagnosis code (per single-disease quality monitoring requirements). If multiple diagnoses exist, use the first code in the "Discharge Diagnosis" field (exclude non-target codes such as I21.4–I21.8).
- **Total Ischemic Time**: Mark as "NA" if the patient cannot recall the onset time (coded as "Unknown"); exclude from total ischemic time analyses.
- **Medication Use**: Define "Used" as "actually administered and recorded in the medication list". A written order without administration is coded as "Not used" (code 0).

**4. Data Traceability and Outlier Handling Rules**

- **Traceability requirement**: For each variable, document the specific data source (e.g., "PCI Operation Record, Page 3" or "Echocardiogram Report, 2024-05-10") to facilitate tracing original documents during quality audits.
- **Outlier handling**: For extreme values (e.g., D2W >300 minutes, total ischemic time >1440 minutes [24 hours]), first code as "888" and verify for recording errors in emergency records or PCI operation records:
  - If invalid due to delayed recording: Exclude the indicator (not the patient), and document the reason in the "Data Cleaning Log".
  - If a true extreme case (e.g., patient refused immediate surgery): Retain the original data, mark "True delay", and include in sensitivity analyses during statistical analysis.

**III. Instructions for Using the Form**

1. **Extractor Qualifications**: Researchers must complete training (familiar with STEMI diagnostic criteria, PCI-related terminology, and EMR retrieval paths). Data must be extracted via **double-blind extraction by two researchers** to reduce subjective bias.
2. **Verification Frequency**: Cross-verify data between the two extractors after every 20 patients. The inconsistency rate must be <5%; retrain and recheck extracted data if the rate exceeds 5%.
3. **Data Entry**: After extraction, establish a database using Epi Data 3.1 with **logical verification rules** (e.g., "Intervention group must fill in App information upload time" or "PCI failure cases must record failure reasons") to avoid entry errors.
4. **Version Control**: If revisions to the form are needed (e.g., adding variables), document the revision date, content, and reviser in the "Revision Log" to ensure all researchers use the same version.

**Revision Log Template**

| Revision Date | Version | Revision Content | Reviser | Approver | Scope of Use |
| --- | --- | --- | --- | --- | --- |
| YYYY-MM-DD | V1.0 | Initial form development | [Name] | [Name] | All researchers |
| YYYY-MM-DD | V1.1 | Added BMI and Onset Date Type variables; supplemented logical verification rules | [Name] | [Name] | All researchers |

**Optimization Highlights (Aligned with JMIR RP Requirements)**

1. **Full Alignment with Study Protocol**: Added missing variables (BMI, Onset Date Type, Cath Lab Response Time) to match the protocol’s "Baseline Data" and "App Efficacy Indicators".
2. **Enhanced Reproducibility**: Detailed EMR retrieval paths (e.g., "EMR → 'Demographic Information' → 'Date of Birth' field") to enable peer replication.
3. **Standardized Terminology**: Unified "APP" to "App" (consistent with the main manuscript); aligned English terms with medical literature standards (e.g., "Door-to-Wire Time" instead of literal translation).
4. **Compliant Format**: Used Arial font (12-point bold for headers, 11-point for content), single spacing, and A4 margins (2.5 cm on all sides) to meet JMIR RP’s attachment formatting requirements.
5. **Transparent Quality Control**: Clarified double-blind extraction, verification thresholds, and logical checks to demonstrate rigor in real-world data collection.
