# Supplementary material for: Impact of a Prehospital Chest Pain Alert App–Mediated Prehospital–in-Hospital Coordination Model on Treatment Delays and Clinical Outcomes in Patients With ST-Elevation Myocardial Infarction: Protocol for a 4-Year Retrospective Real-World Cohort Study
Source: JMIR Res Protoc. 2026 Apr 13;15:e90144. doi: 10.2196/90144 (PMC13075538; doi:10.2196/90144)
Supplement: Multimedia Appendix 4 [file resprot-v15-e90144-s004.PDF]

# 伦理审查批准函

**批件号：** 2025-KY-116-02

| | | | |
|---|---|---|---|
| **项目名称** | 院前胸痛预警 APP 介导的院前-院内协同模式对 STEMI 患者治疗延迟及临床结局的影响：一项基于四年真实世界数据的回顾性队列研究 | | |
| **申办方/组长单位** | 上海市奉贤区中心医院 | | |
| **项目类别** | 研究者发起 | **项目分期** | / |
| **承担科室** | 医疗急救中心 | **项目负责人** | 陈舒元 |
| **审查方式** | 快速审查 | **审查时间** | 2026 年 01 月 13 日 |
| **审查类别** | 初始审查(复审) | | |
| **审查文件** | 详见附件 | | |
| **审查委员** | 侯月梅，李纪文 | | |
| **审查意见** | 1.经本伦理审查委员会审查：批准<br>2.意见和建议：无 | | |
| **跟踪审查频率** | 12 个月 | | |
| **决定生效日期** | 2026 年 01 月 14 日 | | |

上海市奉贤区中心医院伦理审查委员会（盖章）

主任委员/副主任委员签名：

日期：2026.1.14

**注意事项**（请仔细阅读）：

1.本伦理审查委员会人员组成和工作程序符合赫尔辛基宣言的原则，并遵守中国相关法律和法规的规定；

2.研究过程中，如对方案、知情同意书、招募材料等修改，变更项目负责人，须递交修正案审查申请至本委员会，经审查批准后方可执行；

3.本伦理审查委员会对研究项目进行跟踪审查，自批件签发之日起，须在规定的跟踪审查截止日期或批件失效前 1 个月提交项目进展报告；

4.方案偏离、安全性信息、暂停或终止研究、结题、再审等，须按照伦理审查委员会的要求递交本委员会；

5.凡涉及人类遗传资源保护或者按照国家规定必须经有关部门专项审批的内容，均必须在项目执行前向有关部门申报并获得批准；

6.凡经本伦理审查委员会批准的研究项目在实施前，应按规定在医学研究登记备案信息系统登记研究项目的相关信息；

7.本项临床研究应当在伦理审查委员会批准起 1 年内实施；逾期未实施的，本批件自行废止。
